# Let History Be Accurate

**Published:** 1872-08

**Authors:** Geo. Watt


### Let History be Accurate.

*Ed. Dental Register*:

At the late Commencement of the Ohio Dental College, Dr. James Taylor, President of the Board of Trustees, delivered an address to graduates. In this he gives a condensed history of the teachers and teachings of the College. Of the teachers it is mainly eulogistic. I propose to notice a single sentence: "We name also C. B. Chapman, M. D., Professor of Chemistry, a devoted and most excellent teacher."

As it stands thus, in an official, historical document, the reader is left to infer that the only chemical teaching in the College worthy of mention, after that of the sainted Slack, is that imparted by Dr. Chapman. This is not highly complimentary to Drs. Kellogg and A. H. Smith, to say nothing of the undersigned. But the strangest part of all is that Dr. Chapman never lectured on chemistry in the College at all. He was elected to the chemical chair, but went to the war, without filling it. In the session of 1865 and 1866, the Miami Medical College occupied the Dental College Building, and Dr. C. was Professor of Chemistry in that institution; and I hired him to let the dental students hear his lectures on medical chemistry, for a few weeks preceding the holidays. That is the sum total of his chemical teaching in the Dental College. But Dr. Taylor tells the truth (at least I think so) in calling Dr. C. "a devoted and most excellent teacher;" but it was as Professor of *Anatomy* that he figured in the Dental College.

Bad or good, nearly all that has been said about chemistry in the Ohio Dental College, has been feebly spoken by

GEO. WATT.

